# Investigation of Cytotoxic, Antioxidant, Apoptotic/Necrotic Activity of *Aquilaria agallocha* Root Extract and Determination of Gene Expression Levels in HepG2, MCF-7 Cancer Cell Lines

**DOI:** 10.3390/life15040651

**Published:** 2025-04-16

**Authors:** Semih Dalkılıç, Lütfiye Kadıoğlu Dalkılıç, Elgun İsbenov, Lütfü Uygur, Ceydanur Taşdemir

**Affiliations:** Department of Biology and Molecular Biology and Genetics Program, Faculty of Science, Fırat University, 23119 Elazığ, Türkiye; sdalkilic@firat.edu.tr (S.D.); tkadioglu85@gmail.com (L.K.D.); isbenovelgun@gmail.com (E.İ.); tasdemirceyda01@gmail.com (C.T.)

**Keywords:** cytotoxic activity, antioxidant activity, apoptosis, necrosis, expression level

## Abstract

Cancer is a common disease worldwide, and medicinal plants are widely used for its treatment. *A. agallocha* is a plant from the Thymelaeaceae family that is endemic to East Asia. Popularly known as the agar tree, this plant is used in the treatment of heart diseases, asthma, cough, ulcers, gout, inflammation, and pain. In this study, the cytotoxic, apoptotic/necrotic, and antioxidant activities of root extracts of *A. agallocha* and their effects on gene expression were investigated. Cytotoxic effects were analyzed by the MTT assay method, apoptotic/necrotic effects by the double staining method, and antioxidant effects by the DPPH radical scavenging assay. According to the results, the best cytotoxic effect in HepG2 cells was observed at 1000 µg/mL hexane extract. In MCF-7 cells, 250 µg/mL hexane extract showed the best activity. Antioxidant activity was highest in dH2O and lowest in the methanol extract. In gene expression analysis, hexane and methanol extracts decreased p53 expression in HepG2 cells, while acetone extract increased it. In addition, the expression of Bax and Bcl-2 genes generally increased. This study revealed the potent cytotoxic, antioxidant, and apoptotic effects of the *A. agallocha* extract.

## 1. Introduction

Cancer is a disease that occurs as a result of uncontrolled or abnormal growth and proliferation of cells and is usually fatal. The development of cancer is associated with various factors, such as DNA damage, environmental factors, genetic predisposition, dietary habits, and personal lifestyle [1]. This disease is recognized as one of the most serious problems of the 20th century and tends to affect more people throughout the 21st century, with increasing persistence and frequency. The lifelong risk of cancer for one in four people emphasizes the seriousness of this situation [2]. The prevalence and high mortality rates of cancer have made it one of the most critical challenges in modern medicine. By 2040, approximately 29.5 million new cases are expected. This predicts that cancer will rise from second to first place among the causes of death worldwide. Therefore, an effective fight against cancer and early detection are of vital importance [3]. Human breast cancer (MCF-7) is one of the most common types of cancer and is known to cause an increase in cancer-related deaths among women. However, breast cancer remains a universal health problem, with more than 4.4 million women worldwide living with breast cancer and accounting for approximately 31% of all cancers in women [4,5]. Human liver cancer (HepG2), particularly hepatocellular carcinoma (HCC), is the third leading cause of cancer-related deaths in men and the sixth leading cause of cancer-related deaths in women. According to 2002 data, HCCs account for 75–90% of liver cancers, which causes more than 60,000 deaths [6,7]. Overall, 80% of deaths from this cancer have been reported in developing countries, such as Asia and Africa. Many treatment methods have been researched and applied to treat cancer. There are also a number of challenges that prevent cancer treatment from being effective. One example is the development of resistance to various treatment modalities such as targeted chemotherapy and radiation therapy [8]. The tendency of cancer to spread and its treatment can cause functional limitations and other problems in the patients. For example, breast cancer survivors may need rehabilitation and physical therapy to treat lymphedema and post-operative complications [9].

Although plant-derived compounds are used as alternative approaches for cancer treatment, research is ongoing. Studies have shown that a significant proportion of gynecological and breast cancer patients adopt complementary health approaches in addition to medical treatments [10]. Since ancient history, humans have been highly dependent on various plant species for survival. Around 80% of people worldwide rely on traditional medicines to meet their basic health needs. At least 25 percent of drugs used in modern pharmacology are of plant origin [6,11]. According to the World Health Organization, 20,000 plants are used to treat various diseases. One of the plant species used for therapeutic purposes is *Aquilaria agallocha*.

The root of *A. agallocha* belongs to the family Thymelaeaceae and is usually found as an endemic species in East Asian countries (Bangladesh, India, Malaysia, Singapore, and Thailand). It is also popularly known as the agar tree and Ud-i Hindi. The plant, which is usually found at a medium height between 6 and 20 m, is known in many regions such as Gaharu in Indonesia and Malaysia, Aloewood in Hong Kong, Mai Kritsana in Thailand, Mai Ketsana in Laos, Chengxiang in China, and Shajarat-al-oudh in Arabic. This plant is used in conditions such as heart disease, asthma, cough, ulcer, gout, inflammation, and pain. In addition, a study reported that *A. agallocha* seeds contain a fraction rich in phorbol ester, which shows antiallergic properties [12]. *A. agallocha* has anti-neoplastic and immunosuppressive properties. It is a rare tropical rainforest product traded internationally for its distinctive odor. It is not only popular in incense and perfumery but has also become a preferred product in traditional medicine for its sedative, carminative, heart-protective, and pain-relieving effects [13]. *A. agallocha* is a plant species that has been extensively studied for its numerous properties and potential applications. This plant has many advantages, and its essential oil content, medicinal properties, essential oil composition, and genetic characteristics have been extensively studied. The plant has also been found to have antitripanosomal activity [14]. The leaves of the plant are also rich in flavonoids, suggesting that different parts of the plant may have antioxidant properties [15].

## 2. Materials and Methods

### 2.1. Herbal Material

*A. agallocha* roots were purchased commercially from Elazığ province. In addition, plant roots were preserved in the genome science laboratory of Fırat University under appropriate environmental conditions and were used in the experimental study.

### 2.2. Extract Preparation

The root parts of *A. agallocha* were cut into very small pieces. The pieces were dried for 1–2 weeks. After drying, the samples were pounded in a porcelain mortar and powdered. After this procedure, it was weighed on a precision balance, and 1 g was extracted with 10 mL hexane, 10 mL methanol, and 10 mL acetone. After 72 h, the extracts were filtered using Whatman No. 1 filter paper and then dried. Four different concentrations (250 µg/mL, 500 µg/mL, 750 µg/mL, and 1000 µg/mL) were then dissolved in 10 mL dimethylsulfoxide (DMSO), (Sigma-Aldrich, St. Louis, MO, USA) and stored at +4 °C. Hexane, methanol, and acetone solvents were used to obtain the extracts of *A. agallocha*. In addition to these solvents, dH_2_O was also used for antioxidant studies. The extraction process was also performed using purification methods.

### 2.3. Identification of the Components of Phytochemicals

High-Performance Liquid Chromatography (HPLC) is a popular analytical technique for the separation and study of both organic and inorganic materials in a variety of industries, including biological, pharmaceutical, food, environmental, and industrial. Within the scope of this study, the HPLC method was used to quantitatively evaluate the bioactive components in *A. agallocha* extracts. Rutin, myricetin, morin, quercetin, kaempferol, catechin, naringenin, resveratrol, vanillic acid, gallic acid, hydroxycinnamic acid, caffeic acid, ferulic acid, and rosmarinic acid were among the components measured. PREVAIL C18 reversed-phase column (15 × 4.6 mm, 5 µm) (Stockbridge, GA, USA) was used for these studies. The mobile phase was a mixture of methanol/acetonitrile/water (46/8/46, *v*/*v*/*v*) containing 1% acetic acid. Analyses were performed at a flow rate of 1.0 mL/min. Samples were analyzed at 25 °C using a 10 µL injection volume [16].

### 2.4. Cell Culture and MTT Assay

HepG2 and MCF-7 cancer cell lines were obtained from the laboratory of Assoc. Prof. Dr. Semih Dalkılıç, a faculty member of the Molecular Biology and Genetics Program of the Department of Biology at Fırat University. MCF-7 and HepG2 cells were cultured in 75 cm^2^ culture flasks containing Dulbecco’s modified Eagle’s medium (DMEM) (25 mM L-Glutamine, 1% Penicillin-Streptomycin and 10% FBS (Fetal Bovine Serum)) (Gibco™, Langenselbold, Germany) at 37 °C and 5% CO_2_

The MTT assay is a widely used method for assessing cell viability and proliferation. It is based on the reduction of the yellow tetrazolium salt MTT (3-(4,5-dimethylthiazol-2-yl)-2,5-diphenyltetrazolium bromide) to a purple formazan product by mitochondrial enzymes in living cells. It provides a quantitative measure of cell viability and can be used to assess the effects of different treatments or conditions on cell viability. This test is relatively simple and cost-effective, making it a popular choice for many researchers [17].

After the cells grown in 75 cm^2^ flasks reached 90% confluency, the old medium was discarded. These flasks were washed with 5 mL sterile PBS (Phosphate Buffer Solution) (Biosera, France). Next, 1 mL of trypsin-EDTA (Gibco™, Langenselbold, Germany) was added to the flasks and incubated at 37 °C in an oven containing 5% CO_2_ for 2 min. After the cells were detached from the surface, trypsin-EDTA was neutralized with 5 mL of medium. Then, the cells were removed from the flask and centrifuged at 2000 RPM for 5 min; the supernatant was discarded, and the cell pellet was thawed with 1000 µL RPMI. Cell counting was performed using a Countess II automatic cell counter. After calculations were made, cell dilutions were prepared in RPMI, and 96-well plates were seeded with 100 µL RPMI, so that 5 × 10^3^ cells were present in each well. First, only RPMI was added as a blank, 2.5 µg/mL Doxorubicin was used as a positive control, and only medium was used as a negative control. Then, they were incubated at 37 °C in an oven containing 5% CO_2_ for 24 h. After incubation, the medium in the wells was discarded, and four different concentrations of acetone, methanol, and hexane extracts of *A. agallocha* root (250 µg/mL, 500 µg/mL, 750 µg/mL, and 1000 µg/mL) were added to the cells 6 times and incubated in an oven at 37 °C with 5% CO_2_ for 72 h. At the end of the incubation period, 10 μL of MTT solution (5 mg/mL) was added to the cell wells and incubated for 4 h at 37 °C in a dark environment containing 5% CO_2_. The expected color change was then determined by absorbance measurements.

### 2.5. Determination of Apoptotic/Necrotic Activity

Hoechst 33342 andpropidium iodide (PI) dual staining procedure detects apoptotic and necrotic activity to evaluate DNA binding. This technique involves the use of fluorescent dyes that can bind to DNA, thus allowing visualization of chromatin and the cell nucleus. Hoechst 33342 is a fluorescent dye that can bind to DNA because of its ability to pass through the cell membrane. PI dye can only be used by cells with a loss of cell membrane integrity, which can be removed so that advanced apoptotic/necrotic cells can be detected.

MCF-7 and HepG2 cell lines were plated on sterile coverslips and distributed at 10 × 10^3^ cells per 100 µL in each plate. The cells were incubated for 4–5 h to adhere to the coverslip surface. Then, 2 mL DMEM (1% L-Glutamine, 1% Penicillin-Streptomycin, and 10% FBS) was added to the wells and incubated at 37 °C and 5% CO_2_ atmosphere for 24 h. After incubation, the medium in the wells was drained, and 2 mL of medium containing *A. agallocha* extract at 4 different concentrations (250 µg/mL, 500 µg/mL, 750 µg/mL, and 1000 µg/mL) was added and incubated for 48 h. After incubation, 1 mL of a solution prepared with 5–10 µg/mL Hoechst and 1 µg/mL PI dyes (dye solution was prepared in 1x PBS) was added to the wells (1 mL of medium was removed from the well before dye addition). The plate was then incubated in the dark at 37 °C in a 5% CO_2_ atmosphere for 30 min. After incubation, cell morphologies were compared to those of the control groups and evaluated under a fluorescence microscope [18].

### 2.6. Determination of Antioxidant Activity by 2,2-Diphenyl-1-picrylhydrazyl (DPPH) Radical Scavenging Capacity

The DPPH (2,2-diphenyl-1-picrylhydrazyl) assay is a method developed to assess antioxidant activity and is utilized to determine the capability of antioxidants to scavenge free radicals. In this study, the antioxidant activity of *A. agallocha* extracts at various concentrations was evaluated using a DPPH radical scavenging assay. To prepare the samples, lyophilized drug solutions were created from different concentrations of *A. agallocha* extracts dissolved in methanol, hexane, acetone, and distilled water at a concentration of 2.5 mg/mL. These solutions were then diluted threefold and a calibration curve for DPPH was established. Next, the plate containing the samples was incubated in the dark for 30 min to allow the reaction to occur. Subsequently, spectrophotometric absorbance values were measured at 517 nm, and the percentage inhibition values were calculated. The results were then determined using the following formula, adapting it as necessary [19].

%DPPH = (Acontrol − Asample)/Acontrol × 100(1)

This assay provides valuable insights into the antioxidant potential of *A. agallocha* extracts, aiding the assessment of their efficacy in scavenging free radicals.

### 2.7. RNA Isolation and cDNA Synthesis

HepG2 and MCF-7 cell lines were seeded into 6 well plates and incubated at 37 °C with 5% CO_2_ for 24 h. After incubation, cells that reached sufficient confluence were exposed to various concentrations of *A. agallocha* root extract for 48 h. Following this treatment, total RNA isolation was performed to determine the gene expression levels of the cells. The isolation procedure was carried out using the PureLink Total RNA Mini Kit (Invitrogen, Waltham, MA USA) according to the manufacturer’s instructions.

### 2.8. Quantitative Real-Time Polymerase Chain Reaction

Quantitative Real-Time PCR (qRT-PCR) was used to determine the expression levels of Bax, Bcl-2, and p53 genes. This study aimed to quantify the expression levels of apoptotic marker genes. The specific primers used in this study are listed in Table 1. These primers are optimized to ensure the amplification of target genes and are crucial for obtaining reliable and accurate results in qRT-PCR analysis.

GAPDH (glyceraldehyde-3-phosphate dehydrogenase) was used as the endogenous control gene, and untreated cells served as the negative control. Gene expression analyses were conducted using qRT-PCR with SYBR Green master mix (AM02-020) (Blirt, Danzig, Poland) on an Applied Biosystems Step One Plus Real-Time PCR System. The protocol applied was as follows: 10 μL reaction mixture per sample, consisting of 5 μL of 2× Amplifyme SG Universal mix, 0.3 μL of each forward and reverse primer, 0.2 μL of 50× High ROX solution, 2.2 μL of PCR grade water, and 2 μL of cDNA sample. The cycling conditions were as follows: initial denaturation at 95 °C for 3 min, followed by 40 cycles of 95 °C for 10 s, 58 °C for 10 s, and 72 °C for 20 s. Gene expression levels were normalized to those of GAPDH and analyzed using the 2^−ΔΔ^CT method. Data are reported as the mean fold change ± Standard Error of the Mean (SEM) from three independent amplifications.

### 2.9. Statistical Analysis

All statistical analyses were performed using Jamovi and R applications (version 3.6) [20,21,22,23,24]. The statistical difference between the percentages of viable cells was investigated using the Kruskal−Wallis test for each cell line, each extract, and each dosage. Dwass-Steel-Critchlow-Fligner pairwise comparisons were performed post-hoc to understand which dosage, if any, accounted for the statistically significant difference. Regression analysis was performed again to determine if there was a statistically significant relationship between dosage and percentage of viable cells. A violin plot was used to compare between dosage groups. Gene expression levels were calculated by the 2^−ΔΔCT^ method using GAPDH for normalization. Differential expression was investigated for p53, Bax, and Bcl-2 genes compared to the control sample. Differences in gene expression between the extraction methods were also compared. For statistical analysis, the Kruskal−Wallis test was performed for each cell line, each extract, and each dosage. Dwass-Steel-Critchlow-Fligner pairwise comparisons were performed post-hoc to determine which dosage, if any, accounted for the statistically significant difference.

## 3. Results

### 3.1. Quantitative Analysis of Individual Bioactive Components

The first stage of our study was the quantitative analysis of the individual bioactive components of the plant we are studying, *A. agallocha* extract. Component analysis was performed by the HPLC method and the results are shown in Table 2.

Rosmarinic acid (435.67 µg) stood out as the most abundant compound in this analysis. This may play an important role in the antioxidant and anti-inflammatory properties of *A. agallocha* because rosmarinic acid is a potent phenolic compound. Naringin (174 µg) was the second-highest compound. Naringin is a flavonoid and is known for its antioxidant, anti-diabetic, and anticancer properties. Galic Acid (12.67 µg) and Rutin (21.67 µg) were found at intermediate levels. Both are phenolic compounds and are characterized by their antioxidant activity. According to the HPLC analysis results of the *Aquilaria agallocha* plant, compounds such as rosmarinic acid and naringin were dominant. The high concentrations of these compounds indicate that the plant has strong potential antioxidant and anti-inflammatory effects. In addition, flavonoids (such as rutin, kaempferol, and myricetin) and other phenolic compounds suggest that the plant can be evaluated pharmacologically.

### 3.2. Cytotoxic Activity

When the cytotoxic activities of hexane, methanol, and acetone extracts of *A. agallocha* were examined, it was observed that the highest cytotoxic activity on the HepG2 cell line was effective at the highest concentration in hexane and methanol extracts, while different values were observed in the cytotoxic activity in MCF-7 cell line depending on the concentration. In the HepG2 cell line, hexane extract at 1000 µg/mL showed the highest cytotoxic activity with a 71% dead cell count, followed by the methanol extract at 1000 µg/mL with a 69% dead cell count. The acetone extract showed the lowest activity, with 68% at 1000 µg/mL, compared to the extracts with the two best cytotoxic activities. The lowest cytotoxic activity was observed for the methanol extract at a concentration of 250 µg/mL. The hexane extract showed the lowest effect at 250 µg/mL, while this effect was observed in the acetone extract at a concentration of 1000 µg/mL.

**Figure 1:**
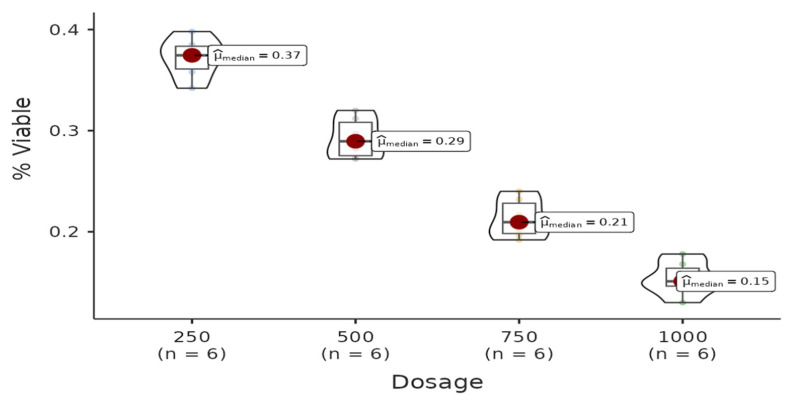
The results of the cytotoxic activity of methanol extracts of *A. agallocha* on HepG2 cell lines are presented.

There was a statistically significant difference among the dosages in the methanol group. All pairwise comparisons among each dosage were found to be significant (W = −4.08, *p* = 0.021 < 0.05). Regression analysis between live cell percentage yielded a model with R^2^ = 0.956 and B(500–250) = −0.08 (*p* < 0.001), B(750–250) = −0.16 (*p* < 0.001), B(1000–250) = −0.22 (*p* < 0.001). Therefore, it can be said that the percentage of live cells decreases with increasing doses of the solution (Figure 1).

**Figure 2:**
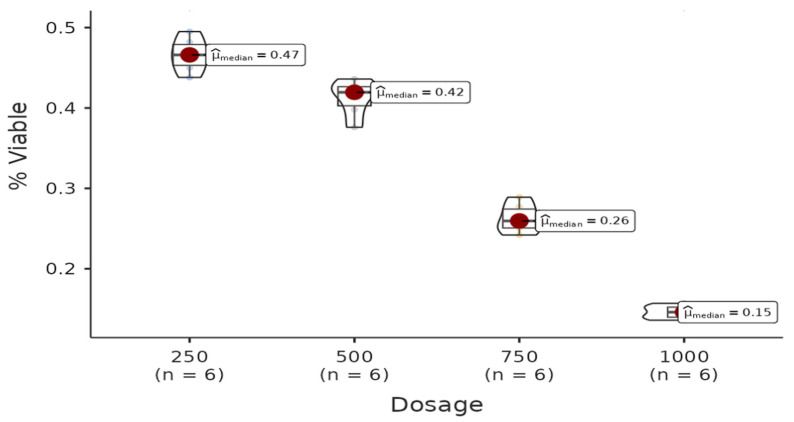
The results of the cytotoxic activity of hexane extracts of *A. agallocha* on HepG2 cell lines are presented. (Positive control (Doxorubicin), negative control (RPMI).)

There was a statistically significant difference among the dosages of the hexane group. All pairwise comparisons among each dosage are found to be significant (W = −4.08, *p* = 0.021 < 0.05). Regression analysis between live cell percentage yielded a model with R^2^ = 0.983 and B(500–250) = −0.05 (*p* < 0.001), B(750–250) = −0.20 (*p* < 0.001), B(1000–250) = −0.32 (*p* < 0.001). Therefore, it can be said that the percentage of live cells decreases with increasing doses of the solution (Figure 2).

**Figure 3:**
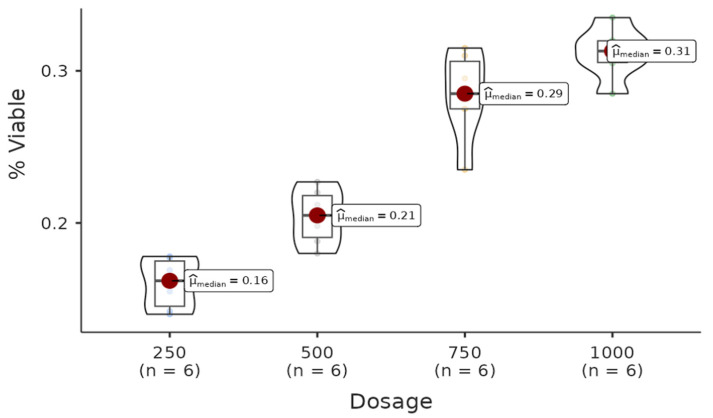
The results of the cytotoxic activity of the acetone extracts of *A. agallocha* on HepG2 cell lines are presented. (Positive control (Doxorubicin), negative control (RPMI).)

There was a statistically significant difference among the dosages in the acetone group. All pairwise comparisons among each dosage were found to be significant (W = −4.08, *p* = 0.021 < 0.05), other than 750–1000 (W = 2.50, *p* = 0.291 > 0.05). Regression analysis between live cell percentage yielded a model with R^2^ = 0.908 and B(500–250) = 0.04 (*p* = 0.002 < 0.05), B(750–250)= 0.12 (*p* < 0.001), B(1000–250) = 0.15 (*p* < 0.001). Therefore, it can be said that the percentage of live cells increases with increasing doses of the solution (Figure 3).

When the cytotoxic activities of hexane, methanol, and acetone extracts of *A. agallocha* were analyzed, as shown in the figure, it was observed that hexane extract showed the best activity on MCF-7 cell line with 88% cell death at 250 µg/mL concentration compared to other extracts. The methanol extract had the best cytotoxic activity, with 51% cancer cell death at a concentration of 1000 µg/mL and the lowest effect at a concentration of 250 µg/mL. The lowest effect in the hexane extract was observed at a concentration of 1000 µg/mL. However, the lowest cytotoxic effect of the acetone extract was observed at a concentration of 500 µg/mL, and the highest cytotoxic effect was observed at a concentration of 1000 µg/mL, with 71% of the cells dead.

**Figure 4:**
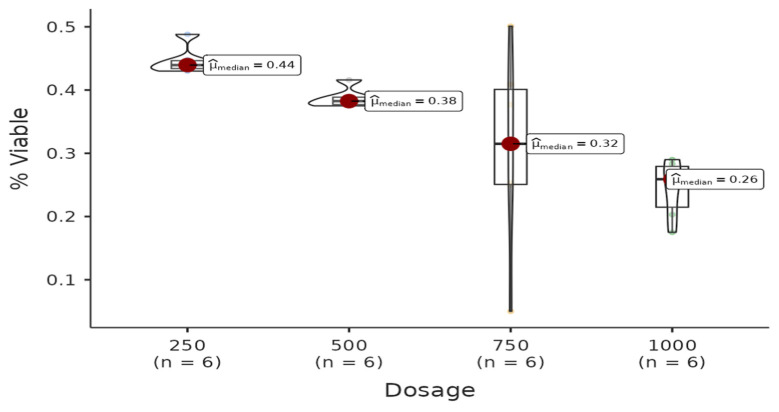
The results of the cytotoxic activity of methanol extracts of *A. agallocha* on MCF-7 cell lines are presented.

There is a statistically significant difference among dosages of the acetone group. All pairwise comparisons among each dosage are found to be significant (W = −4.08, *p* = 0.021 < 0.05). Regression analysis between live cell percentage yielded a model with R^2^ = 0.985 and B(500–250) = −0.17 (*p* < 0.001), B(750–250)= −0.3 (*p* < 0.001), B(1000–250) = −0.42 (*p* < 0.001). Therefore, it can be said that the percentage of live cells decreases with increasing doses of the solution (Figure 4).

**Figure 5:**
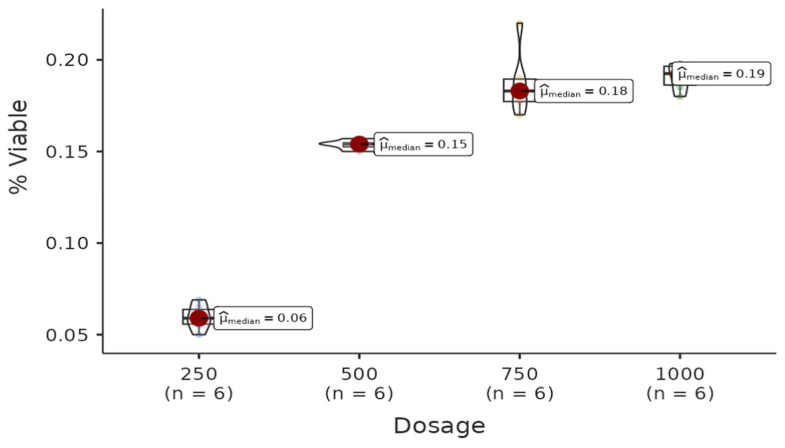
The results of the cytotoxic activity of hexane extracts of *A. agallocha* on MCF-7 cell lines are presented.

There was a statistically significant difference among the dosages in the acetone group. All pairwise comparisons among each dosage were found to be significant (W = −4.08, *p* = 0.021 < 0.05), other than 750–1000 (W = 1.70, *p* = 0.625 > 0.05). Regression analysis between live cell percentage yielded a model with R^2^ = 0.97 and B(500–250) = 0.09 (*p* < 0.001), B(750–250)= 0.13 (*p* < 0.001), B(1000–250) = 0.13 (*p* < 0.001). Therefore, it can be said that the percentage of live cells increases with increasing doses of the solution (Figure 5).

**Figure 6:**
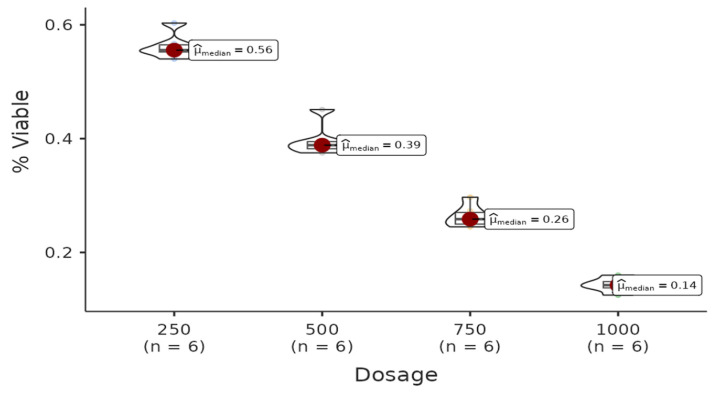
The results of the cytotoxic activity of the acetone extracts of *A. agallocha* on MCF-7 cell lines are presented.

There was a statistically significant difference among dosages of the acetone group. All pairwise comparisons among each dosage are found to be significant (W = −4.08, *p* = 0.021 < 0.05). Regression analysis between live cell percentage yielded a model with R^2^ = 0.985 and B(500–250) = −0.17 (*p* < 0.001), B(750–250)= −0.3 (*p* < 0.001), B(1000–250) = −0.42 (*p* < 0.001). Therefore, it can be said that the percentage of live cells decreases with increasing doses of the solution (Figure 6).

Cytotoxic activity results of *A. agallocha* extracts were obtained by calculating the maximum inhibitory concentration (IC_50_) values for four different concentrations (1000, 750, 500, and 250 μg/mL). IC50 values were calculated for both cell lines and three extract types, and the results are presented in table (Table 3). These results showed variability depending on the cell line and extract type used.

### 3.3. Double Staining (Hoechst 33342 and Propidium Iodide) Method

**Figure 7:**
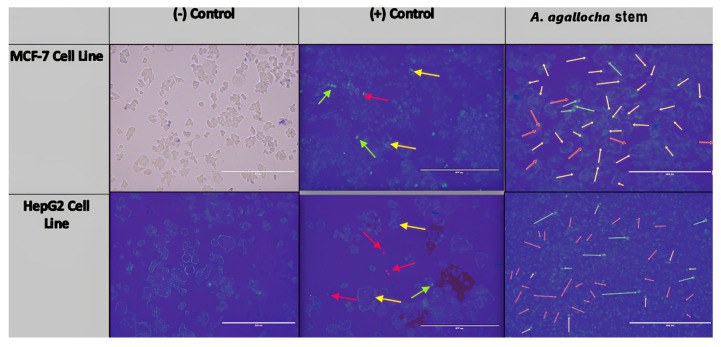
Double staining results. (Yellow arrows indicate apoptosis formation, red arrows indicate necrosis formation, and green arrows indicate chromatin condensation (positive control: Doxorubicin and cancer cell line, negative control: RPMI and cancer cell line).)

Images of methanol extracts of *A. agallocha* after 48 h of treatment of MCF-7 and HepG2 cell lines are presented (Figure 7). In these images, Hoechst and PI fluorescent dyes were used to examine the nuclear morphology and membrane integrity of the cells. The Hoechst stain can stain living and dead cells, while the PI stain can only stain membrane-damaged cells. Typical features of apoptosis include chromatin condensation and pyknosis formation, indicating that pyknosis is a defining marker of apoptotic cells. Likewise, indicators of apoptotic morphology include cell shrinkage and vesicle formation. Since the membrane integrity of apoptotic cells is preserved, they do not stain or stain less with the PI stain, indicating that these cells have died due to apoptosis. Necrotic cells usually have contiguous and swollen cell shapes and may have damaged membranes. They can be stained with both stains, although the hallmark stain for necrotic cells is PI. In all cancer cell lines used, untreated negative (−) control cells remained healthy and increased in number. Hoechst dye stained the cells in the negative (−) control group because it can penetrate all membranes, while PI dye, which can stain membrane-damaged cells, was not effective in this group. According to these results, the cells maintained their viability and proliferated, as expected. In the positive (+) control group, the number and density of cells decreased, and they were generally stained with PI stain. This shows that Doxorubicin kills the cells through necrosis.

Methanol extract applied to the HepG2 cell line caused more necrosis of the cells but also caused partial apoptosis of the cells. In MCF-7 cells, it was observed that the methanol extract applied to the positive control group cells resulted in a more apoptotic cell-dominated image.

### 3.4. Antioxidant Activity

The antioxidant activity of *A. agallocha* was determined by examining the free radical scavenging effect using the DPPH method. As shown in Table 4, the plant had the best radical scavenging effect of 42.01% in dH_2_O compared to other solvents (Table 4). The hexane extract showed the second-best antioxidant effect, with a rate of 40.79%, while the lowest antioxidant activity was observed in the methanol extract, with a scavenging effect of 37.98%. The acetone extract was found to have a 40% free radical scavenging effect.

### 3.5. Expressions of Bcl-2, p53 and Bax Genes

In this study, HepG2 and MCF-7 cell lines were treated with methanol, hexane, and acetone extracts of *A. agallocha* roots at doses of 1000, 750, 500, and 250 μg/mL for 48 h. After incubation, RNA samples were isolated, and the most concentrated and high-quality samples were used to determine the expression levels of the p53, Bcl-2, and Bax genes.

Below, we evaluate the expression levels of Bax, Bcl-2, and p53 genes in the HepG2 cell line by comparing four different concentrations of extracts prepared from *A. agallocha* root using methanol, hexane, and acetone solvents. This evaluation comprehensively examines the effects of different solvents on gene expression levels. *A. agallocha* extracts prepared with different solvents show variability in their effects on the expression levels of Bax, Bcl-2, and p53 genes.

Acetone extracts significantly increased the expression of both Bax and p53 genes, while there was a significant increase in Bcl-2 gene expression. Methanol extracts suppressed the expression of Bax and p53 genes but increased Bcl-2 gene expression. Hexane extracts showed a slight increase in Bax gene expression, suppression of p53 gene expression, and an increase in Bcl-2 gene expression. These results indicate that *A. agallocha* root extracts may affect gene expression differently depending on the solvent used. However, when statistical analysis was performed with the expression raw data of *A. agallocha* plant roots, no significant results were obtained. Although this may be due to the number of samples used in the study, the study should be repeated with a larger sample size for clearer results.

**Figure 8:**
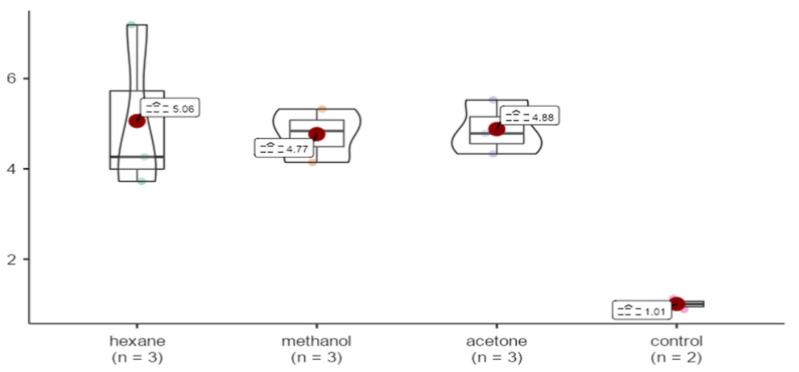
Effects of extracts obtained from methanol, hexane, and acetone solvents on p53 expression in *A. agollacha* roots at 1000 μg/mL concentration in HepG2 cell line.

All samples were observed to be around 4-fold upregulated in comparison to GAPDH. No statistically significant differences were detected for p53 expression levels among the different extraction methods. (X^2^ = 4.74, *p* = 0.192 > 0.05) (Figure 8).

**Figure 9:**
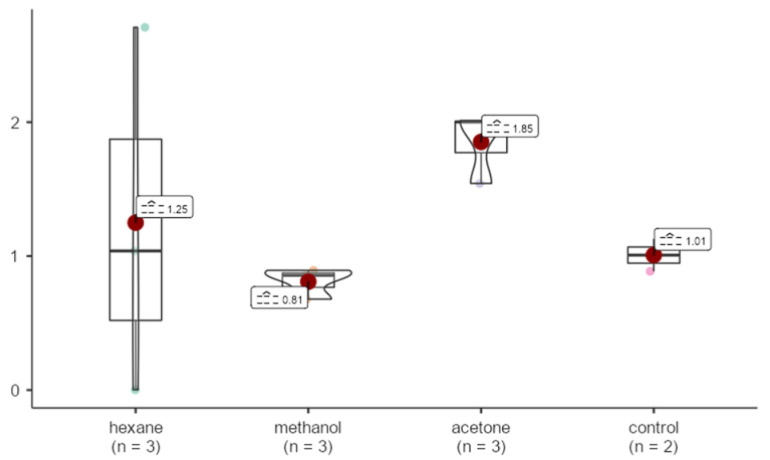
Effects of extracts obtained from methanol, hexane, and acetone solvents on Bax expression in *A. agollacha* roots at 1000 μg/mL concentration in HepG2 cell line.

Hexane and acetone samples were upregulated in comparison to GAPDH by 1.25 and 1.85 fold, respectively. Bax gene expression in the methanol sample was %19 decreased than that of GAPDH. No statistically significant differences were detected in Bax expression levels among the different extraction methods. (X^2^ = 4.44, *p* = 0.218 > 0.05) (Figure 9).

**Figure 10:**
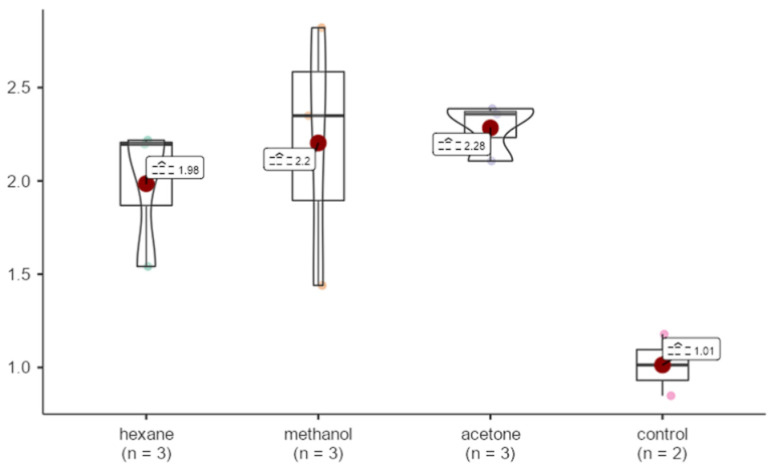
Effects of extracts obtained from methanol, hexane, and acetone solvents on Bcl-2 expression in *A. agollacha* roots at 1000 μg/mL concentration in HepG2 cell line.

A (~) two-fold increase was observed among all samples in comparison to GAPDH. No statistically significant differences were detected in Bcl-2 expression levels among the different extraction methods. (X^2^ = 5.29, *p* = 0.152 > 0.05) (Figure 10).

**Figure 11:**
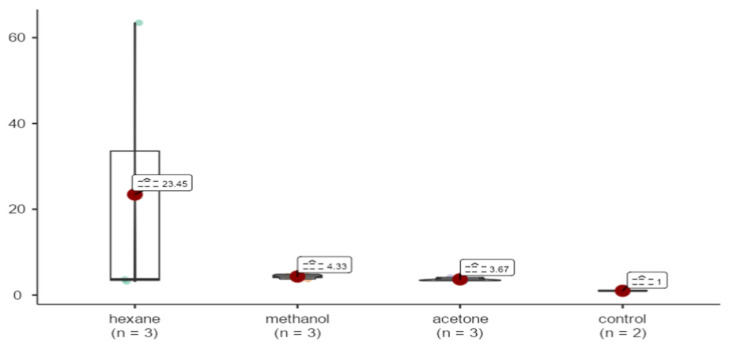
Effects of extracts obtained from methanol, hexane, and acetone solvents on p53 expression in *A. agollacha* roots at 1000 μg/mL concentration in MCF-7 cell line.

p53 gene expression in the hexane sample was observed to be 23.45 fold upregulated in comparison to GAPDH. Methanol and acetone samples were upregulated 4.33 and 3.67 fold. No statistically significant differences were detected in p53 expression levels among the different extraction methods. (X^2^ = 5.77, *p* = 0.123 > 0.05) (Figure 11).

**Figure 12:**
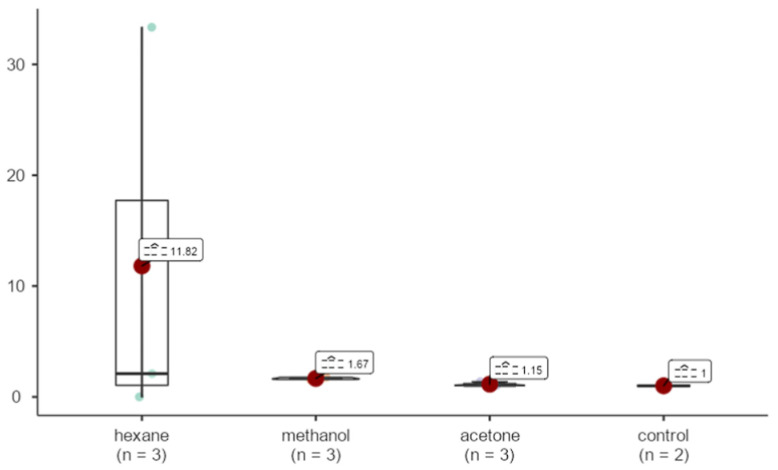
Effects of extracts obtained from methanol, hexane, and acetone solvents on Bax expression in *A. agollacha* roots at 1000 μg/mL concentration in MCF-7 cell line.

Bax gene expression in the hexane sample was 11.82 fold upregulated than that of GAPDH. Methanol and acetone samples were upregulated by 1.67 and 1.15 fold. No statistically significant differences were detected for BAX expression levels among the different extraction methods. (X^2^ = 3.47, *p* = 0.325 > 0.05) (Figure 12).

**Figure 13:**
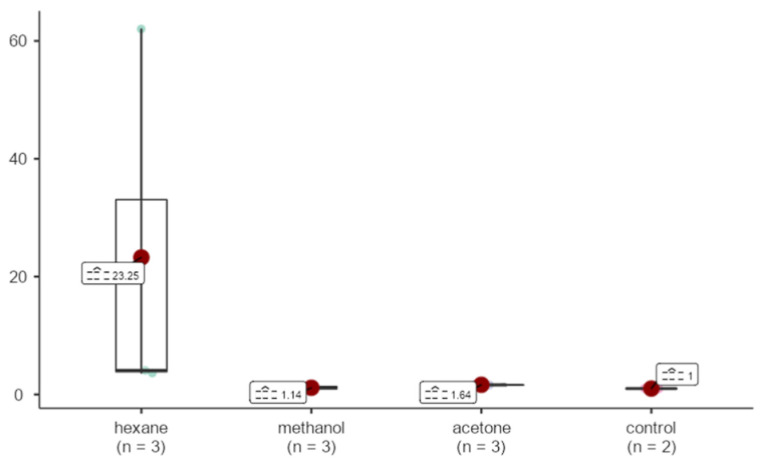
Effects of extracts obtained from methanol, hexane, and acetone solvents on Bcl-2 expression in *A. agollacha* roots at 1000 μg/mL concentration in MCF-7 cell line.

Bcl-2 gene expression in the hexane sample was observed to be 23.25 fold upregulated in comparison to GAPDH. Methanol and acetone samples were upregulated by 1.14 and 1.64 fold. There was an overall statistically significant difference among groups (X^2^ = 8.80, *p* = 0.032 < 0.05), but no single pairwise comparison yielded a significantly different result (Figure 13).

## 4. Discussion

In traditional medicine, herbal therapy is favored as a common practice in the treatment of cancer with medicinal plants, such as *A. agallocha*. A study of the extracts obtained from the root parts of *A. agallocha* showed that this plant has anticancer, apoptotic/necrotic, and antioxidant activities. Cytotoxic activities in HepG2 and MCF-7 cell lines were examined, and it was determined that hexane extract had the best effect [25]. This may reveal the role of this plant in cancer. A study on plant extracts obtained from *A. agallocha* and *Aquilaria malaccensis* revealed that they showed anticancer properties in lung adenocarcinoma cells through activation of the signaling pathway [26]. The essential oil of *A. agallocha* was subjected to analysis, and several compounds were identified through the use of gas chromatography-mass spectrometry. It is hypothesized that these compounds possess anticancer properties [27].

The protective effects of *A. agallocha* extract against paracetamol-induced liver toxicity were studied. The results of the study demonstrated the protective effects of *A. agallocha* against liver damage, revealing the potential of this plant as a natural remedy for conditions such as certain types of cancer, including liver-related diseases [28]. In an antioxidant study of *A. agallocha* with ethyl acetate, it was reported that the plant exhibited high antioxidant activity at concentrations of 500 to 3500 µg/mL and also exhibited pro-oxidant activity with increasing concentrations [29]. The antioxidant activity and phenolic content of *A. agallocha*-extracted chitosan edible films were also measured, and it was observed that this activity increased with increasing concentration and physical properties of the films [30]. At 1000 μg/mL, *A. agallocha* suppressed the proliferation of A549 (lung cancer cell line) cells by 50–55%, whereas the viability of A549 cells was decreased by *A. agallocha* in a dose-dependent manner. The results of this study showed that *A. agallocha* is safe for non-cancerous cells and is significantly toxic to cancer cells [26]. The results of the antioxidant activity study on *A. malaccensis* in Indonesia showed that the antioxidant effect of the plant from Laru and the antioxidant effect of the plant from Hutanabolon were very strong [31]. When the paracetamol-induced hepatotoxicity of ethanolic extracts of plant leaves was examined in SD rats, it was proven that *A. agallocha* has protective properties and hepatoprotective effects [28]. In the study titled Determination of antioxidant, anticancer, and apoptotic activity of β-caryophyllene, an essential oil of *A. crassna* plant, it was observed that β-caryophyllene exhibited good antioxidant activity and showed a strong inhibition against clonogenicity, migration, invasion, and spheroid formation against colon cancer cells. It has also been found to induce apoptosis through a number of distinct pathways, including nuclear condensation, fragmentation, and disruption of mitochondrial membrane potential [32]. According to the study, *A. malaccensis* was observed to contain significantly higher amounts of flavonoids and phenolic compounds compared to *A. agallocha*. However, flavonoids are thought to play an important role in preventing cell damage [26,33]. In a study conducted to reveal the antioxidant properties of *A. agallocha*, it was found that the ethyl acetate extract treated with the leaves of the plant showed the best antioxidant effect at the lowest concentration and the lowest effect at the highest concentration, considering concentrations of 500, 1000, 1500, and 2000 µg/mL [34]. In another study, the antioxidant activity of *A. agallocha* was investigated using the DPPH radical. The results of DPPH radical scavenging activity showed the presence of antioxidant active substances in the soluble parts of 80% methanol, dichloromethane, and ethanol extracts of *A. agallocha* [35]. In addition to the antioxidant and anticancer properties of *A. agallocha*, its antimicrobial effects have been investigated. In one study, the in vitro antimicrobial activity of *A. agallocha* was investigated by extracting the plant roots with an ethanol solvent. The effect of *A. agallocha* on 17 different bacteria and one fungal species showed that the plant had good antimicrobial activity and showed the clearest activity on *E. coli* ATCC 25922 and *Salmonella typhimurium* SL 1344 [36]. In the MTS test applied to *Excoecaria agallocha*, which belongs to the same family as *A. agallocha*, it was determined that it had the highest cytotoxic activity at 4 µg/mL and 7 µg/mL in Capan-1 and Miapaca-2 pancreatic cancer cells [37]. Studies have also shown that this plant has antioxidant activity [38]. The results of the studies reported in the review showed that 57% of the 154 new compounds identified from *Aquilaria* plants were 2-(2-phenylethyl)-4H-chromen-4-one derivatives and 35% were sesquiterpenes. Most of these new compounds, 89%, were isolated from *A. sinensis* [39]. In another study, it was also determined that the essential oil obtained from Agallocha agarwood compounds has an inhibitory role against oxidative damage caused by hydrogen peroxide (H_2_O_2_) according to a test on PC12 (mouse adrenal medullary chromaffin cells) cells [40]. In a separate study, the effects of an aqueous extract of *A. agallocha* Roxb on immediate hypersensitivity reactions were investigated. The aqueous extract showed inhibitory effects on passive cutaneous anaphylaxis, anaphylaxis induced by compound 48/80, and histamine release from rat peritoneal mast cells (RPMC), and morphological examination clearly showed that the extract prevented degranulation of RPMC in rats.

These results suggest that the aqueous extract of *A. agallocha* roots inhibits immediate hypersensitivity reactions by inhibiting histamine release from mast cells [41]. In a gene expression study, in vitro and in silico experiments with *A. agallocha* and *A. malaccensis* extracts showed that these plants increased the expression of Bax, Casp 3, Casp 9, and p53 genes on A549 (Human Lung Carcinoma). In the same study, it was observed that the Bcl-2 gene is downregulated via the intrinsic mitochondrial pathway [26]. According to a study conducted with *E. agallocha* in A549 and H1299 (human lung cancer) cell lines, western blot analysis of Bcl-2, Bax, p53, p21, and PARP (Poly (ADP-Ribose) Polymerase) gene expression revealed that p21 was significantly upregulated in both cell lines, and apoptosis of A549 cells led to significant upregulation of the p53 gene. After treatment of A549 cells, the Bcl-2 expression level showed a dose-dependent decrease, and Bax levels remained unchanged at the basal level in both cell lines [37].

## 5. Conclusions

In this study, the cytotoxic, apoptotic/necrotic, and antioxidant activities as well as the gene expression levels of extracts obtained from the root parts of *A. agallocha* were investigated. These results indicate that the plant exhibits significant cytotoxic effects on MCF-7 and HepG2 cell lines. Additionally, it was found that apoptosis and necrosis are involved in cell death mechanisms, supporting the potential therapeutic effects of the plant against cancer cells. Antioxidant activity studies also show that *A. agallocha* extracts effectively scavenge free radicals. Furthermore, the impact on apoptotic marker genes in HepG2 and MCF-7 cancer cell lines suggests that these findings could support advancements in cancer research.

Although the gene expression levels between samples were not statistically significant in most cases, it is important to note that the sample sizes were small. For the hexane samples in MCF-7 cell lines, both the high range in gene expression levels and the high mean score can be interpreted as interesting.

The findings of this study demonstrate that *A. agallocha* root extract can exhibit significant biological effects on cancer cells and can be considered a potential anticancer agent. It is anticipated that this study will contribute to the development of new and effective herbal treatment options for cancer therapy. These results support the use of this plant as a potential natural resource for the treatment of cancer and other health problems. Future studies can further support these positive findings by further investigating the biochemical components and possible mechanisms of this plant. This suggests that *A. agallocha* may play an important role in the development of new therapeutic modalities in the pharmaceutical and medical fields.

## Figures and Tables

**Table 1:** Primers used in the evaluation of expression profiles.

Primer	Base Sequence (5′-3′)
**GAPDH R**	GAA GAT GGT GAT GGG ATT TC
**GAPDH F**	GAA GGT GAA GGT CGG AGT C
**Bax R**	GAG CTA GGG TCA GAG GGT CA
**Bax F**	CCA CGA TTC ATC TAC CCT GC
**Bcl-2 R**	CTG TGT TGA ACA GGC CAC G
**Bcl-2 F**	GAA GGT TTC CTC GTC CCT GG
**p53 R**	CAT CCA AAT ACT CCA CAC GCA A
**p53 F**	GCT GCT CAG ATA GCG ATG GTC T

**Table 2:** HPLC results of *A. agallocha*.

Compound	Amount (µg)
Gallic Acid	12.67
Vanillic Acid + Caffeic Acid	5
Rosmarinic Acid	435.67
Catechin	10.48
Naringin	134
Rutin	21.67
Myricetin	6
Naringenin	28
Kaempferol	3.33

**Table 3:** IC_50_ value of cytotoxic activity observed in cells.

**MCF-7 Cell Line**			
**Agent**	*A. agallocha* hexane extract	*A. agallocha* methanol extract	*A. agallocha* acetone extract
**IC_50_**	467.6715 μg/mL	719.8459 μg/mL	734.9562 μg/mL
**HepG2 Cell Line**			
**Agent**	*A. agallocha* hexane extract	*A. agallocha* methanol extract	*A. agallocha* acetone extract
**IC_50_**	753.9799 μg/mL	733.3376 μg/mL	1102.999 μg/mL

**Table 4:** The radical scavenging effects of hexane, water, methanol, and acetone extracts of *A. agallocha* root were determined by the %DPPH method.

Concentrations	Solution %DPPH Reduction			
	Methanol	Hexane	Acetone	dH_2_O
**12.5 µg/mL**	0.4 ± 1.3	0.50 ± 0.121	0.5 ± 7	0.0005 ± 0.0013
**25 µg/mL**	0.95 ± 0.75	1.01 ± 0.631	1 ± 6.5	0.001 ± 0.0008
**50 µg/mL**	1.9 ± 0.2	0.002 ± 0.377	2 ± 5.5	0.002 ± 0.0002
**100 µg/mL**	3.8 ± 2.1	0.004 ± 0.375	4 ± 3.5	0.004 ± 0.0022

## Data Availability

The data contained in this article are part of the research project.
